# A Case Series of Secondary Aortoenteric Fistula after Open Aortic Aneurysm Repair: Timing and Technique of Surgery

**DOI:** 10.3400/avd.cr.22-00031

**Published:** 2022-12-25

**Authors:** Kentaro Kasa, Hiroshi Hirukawa, Shintaro Fukuda, Fuyuki Asami, Masatake Katsu, Kazuo Yamamoto, Shinpei Yoshi

**Affiliations:** 1 Department of Surgery, Tachikawa General Hospital, Nagaoka, Niigata, Japan; 2 Division of Vascular Surgery, Department of Surgery, The Jikei University School of Medicine, Tokyo, Japan; 3 Department of Cardiovascular Surgery, Tachikawa General Hospital, Nagaoka, Niigata, Japan

**Keywords:** secondary aortoenteric fistula, graft aortoenteric fistula, graft aortoenteric erosion

## Abstract

Secondary aortoenteric fistula (sAEF) is a rare but serious complication after open aortic aneurysm repair (OAR). Although there is no consensus on the treatment strategy for sAEF, acute management of bleeding and infection control greatly affect the outcome. We report five cases of sAEF following OAR from 2016 to 2021. One patient died of sepsis following graft infection, whereas the others had relatively good outcomes. No recurrence of infection or fistula has been observed over an average follow-up period of 29.8 months. Timely management of bleeding and infection with surgical intervention resulted in favorable outcomes in our patients.

## Introduction

Secondary aortoenteric fistula (sAEF) is a rare but serious complication after open aortic repair (OAR) of aneurysms with an incidence rate of 0.36% to 1.6%^1)^ and a mortality rate of 13% to 57%.^2)^ The pathology of sAEF is classified as graft aortoenteric fistula (AEnF) and graft aortoenteric erosion (AEnE). AEnF has true fistulous communications between the intestine and the graft, whereas AEnE has communication between the intestine and the external surface of the graft without actual fistulation, mostly developing secondary to infection. AEnE is considered a preliminary step in the process that ultimately leads to AEnF formation.^3)^ By definition, the presence of a fistula demonstrated in pathological analysis differentiates two diagnoses. However, those are often differentiated clinically in their early stage of presentation. Patients with AEnF often present with rapid onset of pain with a sign of gastrointestinal bleeding,^1)^ whereas those with AEnE present with gradual onset of malaise or anorexia often accompanied by fever. There is currently no consensus on the treatment strategy for AEnF and AEnE, although management of bleeding and infection in the acute perioperative phase is the key factor to determine prognosis. We report five cases of sAEF after OAR from 2016 to 2021.

## Case Report

Patients’ data were obtained retrospectively from the medical records of Tachikawa General Hospital, Niigata, Japan. The need for ethics committee approval was waived because of the retrospective nature of the study. All patients provided consent for publication of their case details. The patients’ demographic data are shown in **Table 1**. Their mean age was 73.2 years (range: 70 to 76 years). Primary surgeries were conducted for abdominal aortic aneurysm at our hospital, one of which was mycotic aneurysm. The chief complaint of the patients with AEnF was hematemesis due to the fistula, and the chief complaints of the patients with AEnE were fever and anorexia due to infection. Computed tomography of all cases showed air around the graft, which is a typical finding seen in sAEF cases. Fistulae and/or erosions were formed in the horizontal part of the duodenum in all five patients. They did not have any infectious source other than the aortic lesion in all five patients.

**Table 1:** Table 1 Characteristics, details, and outcomes of the five cases

	Case 1	Case 2	Case 3	Case 4	Case 5
Age (years)	76	75	70	70	75
Sex	Male	Male	Male	Male	Female
Chief complaint	Hematemesis	Hematemesis	Hematemesis	Fever	Anorexia
Medical history	HT	HT, DL, DM, CKD, AP	HT, DL, AP	HT, SSS (p-PCI)	IE, Cerebral Infarction, AF, HT, HUA
Operation history	Juxta-renal AAA replacement	Infra-renal AAA replacement	Infra-renal AAA replacement	Infra-renal AAA replacement	Infra-renal mycotic AAA replacement
Duration from the primary surgery (year)	6.5	2.75	5	3.3	1.5
Pathology	AEnF	AEnF	AEnF	AEnE	AEnE
1st intervention	Partial graft replacement (Reconstruction of CeA/SMA/RA), Partial duodenectomy	EVAR	EVAR	Partial graft replacement with rifampicin-soaked graft, Partial duodenectomy	Partial graft replacement with rifampicin-soaked graft, Partial duodenectomy
Interval until radical surgery (day)	0	86	32	35	20
Detected bacteria	*Streptococcus anginosus*	*Granulicatella adiacens*, *Staphylococcus epidermis* *MRS, *Corynebacterium* spp., *Enterococcus faecium*	*Staphylococcus aureus* *MRSA	*Staphylococcus epidermis* *MRS, *Leuconostoc* species	*Olsenella uli*, *Slackia exigua*, *Atopobium parvulum*
Detected fungus	—	*Candida albicans*	—	*Candida albicans*	—
Intravenous antibiotics	TAZ/PIPC	MEPM, VCM	—	TAZ/PIPC, MEPM	MEPM, VCM
Oral antibiotics	AMPC	—	LZD, ST	AMPC	—
Antifungal drug	—	MCFG	—	MCFG, FLCZ	—
Complication	Cholecystitis Duodenum sutural insufficiency	Graft infection	None	None	Intramuscular abscess
Length of stay (day)	59	150	110	82	35
Outcome	Survived	Died	Survived	Survived	Survived
Cause of death	—	Sepsis	—	—	—

HT: hypertension; DL: dyslipidemia; DM: diabetes mellitus; CKD: chronic kidney disease; AP: angina pectoris; SSS: sick sinus syndrome; p-PCI: postpercuta- neous coronary intervention; IE: infective endocarditis; AF: atrial fibrillation; HUA: hyperuricemia; AAA: abdominal aorta aneurysm; AEnF: aortoenteric fistula; AEnE: aortoenteric erosion; CeA: celiac artery; SMA: superior mesenteric artery; RA: renal artery; EVAR: endovascular aortic repair; *MRS: methicillin-resistant staphylococci; MRSA: methicillin-resistant staphylococcus aureus; TAZ/PIPC: tazobactam/piperacillin; MEPM: meropenem; VCM: vancomycin; DAP: daptomycin; TEIC: teicoplanin; LZD: linezolid; AMPC: amoxicillin; ST: sulfa/trimethoprim; MCFG: micafungin; FLCZ, fluconazole

AEnF patients underwent emergency surgical intervention, whereas AEnE patients underwent open surgery after a course of antibiotic treatment. All patients eventually underwent radical surgery. One AEnF patient underwent emergency radical surgery and two AEnF patients underwent emergency endovascular abdominal aortic aneurysm repair (EVAR) for hemostasis. As for patient case 1, no EVAR specialist was available in our region at that time and the decision was made to proceed open surgery instead of risking his life by waiting or transferring him. Considering their poor general condition, one of the two AEnF patients who had emergency EVAR underwent gastro-jejunal bypass to avoid invasive surgery. However, because peri-fistula infection was observed later, he eventually underwent radical surgery. Radical surgery was performed on a mean of 34.6 days (range: 0 to 86 days) after admission. Pathological analysis indicated that AEnF patients had a fistula whereas AEnE patients did not have a fistula. However, the cause of the fistula was not identified by pathological analysis in any of the patients.

**Figure 1:**
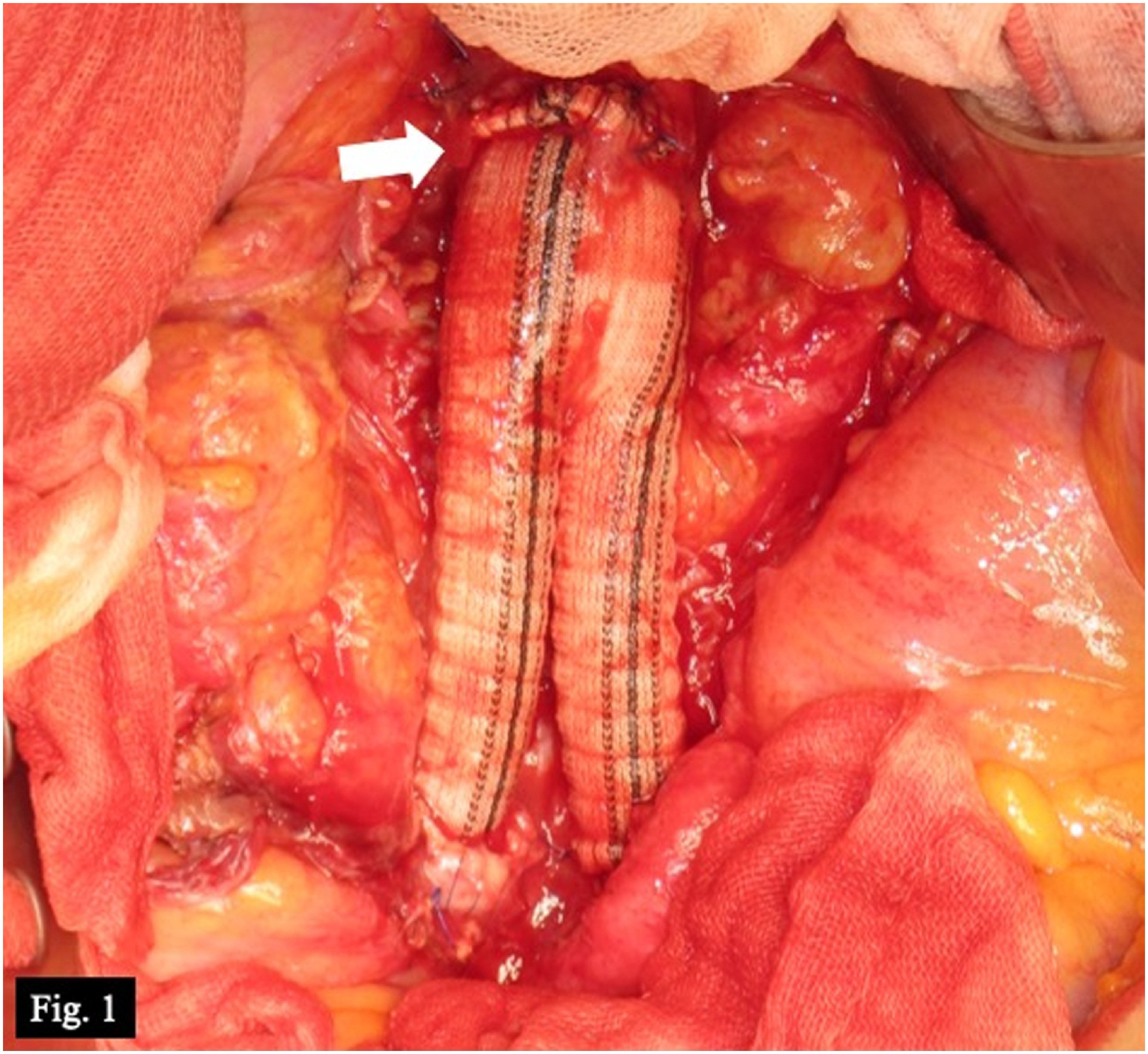
Fig. 1 Operative procedure of in situ reconstruction with a rifampicin-soaked graft (case 5). Central (arrow) and peripheral anastomoses.

**Figure 2:**
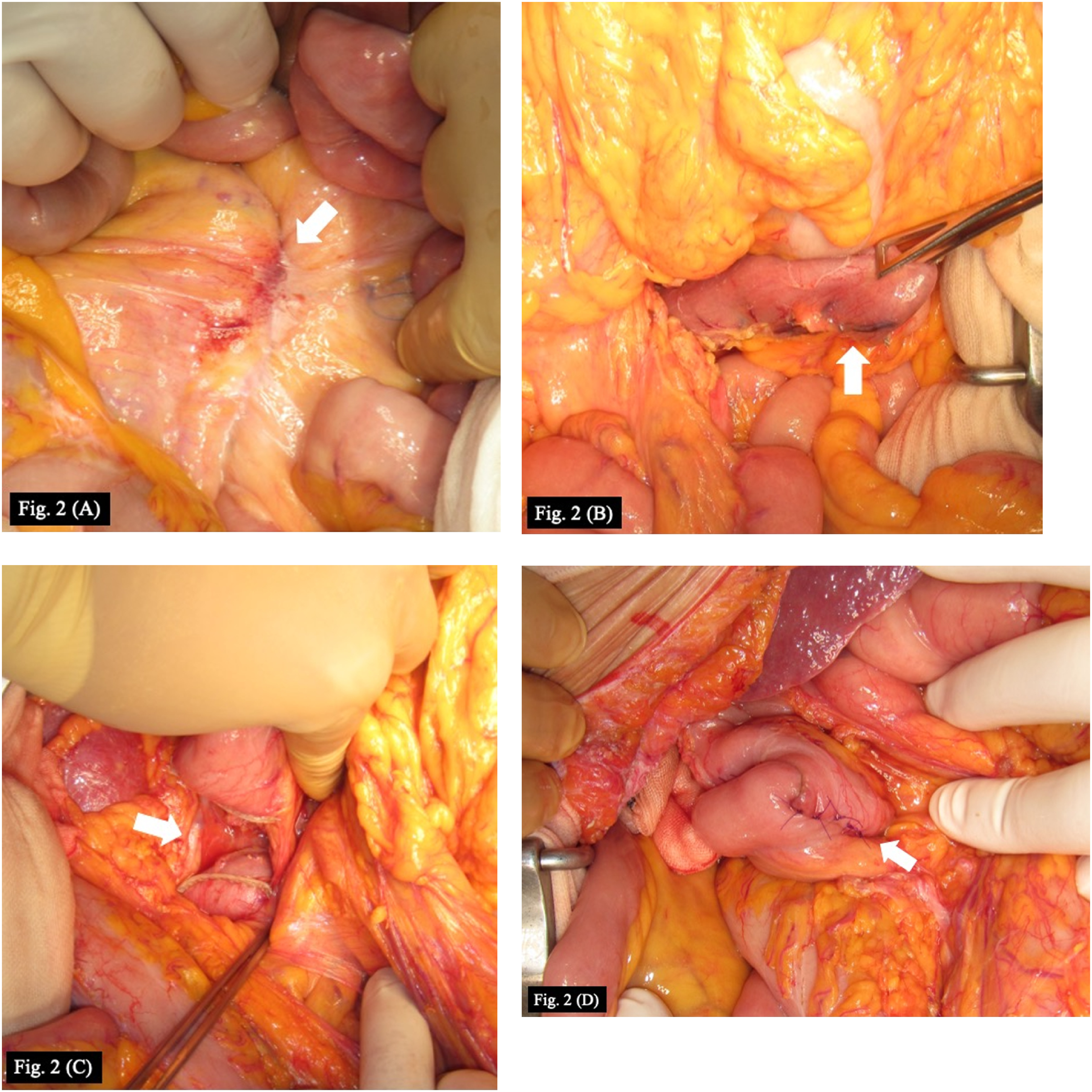
Fig. 2 Operative steps of enteric isolation and anastomosis (case 5). (**A**) Fistula between the prosthetic graft and duodenum, which formed a mass (arrow). (**B**) Resection of the jejunum. (**C**) Resection of the duodenum. (**D**) Side-to-side anastomosis of the duodenum and jejunum.

We performed in situ reconstruction (ISR) by insertion of an aortic vascular graft and partial duodenectomy. We chose partial replacement of the aorta instead of complete removal of the existing grafts for all patients due to extensive adhesions between the grafts and surrounding tissues (**Fig. 1**). Rifampicin-soaked prostheses were used in three cases. The stent grafts that had been previously implanted during EVAR were completely removed. The bowel was repaired with side-to-side anastomosis (**Fig. 2**) or functional end-to-end anastomosis of the remaining duodenum and jejunum.

The patients’ postoperative courses are shown in **Table 1**. One patient developed postoperative infection of the new graft and died of sepsis. The course of another patient was complicated by anastomotic leakage and acute cholecystitis, both of which resolved with conservative management with antibiotics. One patient had a preoperative abscess in the adductor femoris muscle, which resolved with surgical drainage and antibiotics. The four surviving patients were discharged from the hospital. No complications, including recurrence or infection, were observed over the average follow-up period of 29.8 months (range: 1.0 to 62.6 months) after surgery.

## Discussion

The pathogenesis of sAEF is either a pseudoaneurysm at the anastomotic site of the graft or the graft itself mechanically compressing the adjacent intestine.^4)^ AEnF begins with the formation of a pseudoaneurysm secondary to infection or hematoma around the anastomosis, resulting in a fistulous communication between the graft and the intestine. Patients usually present with bleeding, as shown in our report. AEnE occurs because of the pulsatile pressure of the graft causing intestinal wall collapse and leakage, resulting in local inflammation and adhesion. AEnE might progress to AEnF, with the progressive local inflammation leading to the formation of a fistulous communication between the aorta and the intestine. In this report, AEnF patients presented with hematemesis, whereas AEnE patients presented with fever or anorexia.

There are various proposals for the treatment of sAEF in multiple case reports, although no evidence-based consensus has been established yet. The main points discussed in these reports included the following: (1) the two-stage strategy consisting of EVAR for acute hemostasis, followed by radical surgery, (2) the timing of radical surgery, and (3) the resection of the fistulous part of the aorta and the revascularization technique.

There are multiple reports of the two-stage strategy.^5–7)^ In each report, acute hemorrhage was treated using EVAR, although the authors proposed different methods for the radical surgery in the second stage. We adopted the two-stage strategy without resecting the fistulous aorta during the radical surgery in case 2. In this case, although hemodynamic stability was achieved by EVAR, the infection was poorly controlled and the patient eventually died of sepsis. In case 3, we adopted the two-stage strategy with the resection of the fistulous aorta and EVAR graft at the second stage and achieved a favorable outcome. When a patient is in a state of hemorrhagic shock due to AEnF, initial management with EVAR as part of the two-stage strategy is highly effective for promptly achieving hemodynamic stability without the development of infection.

In terms of the timing of radical surgery, there are some reports of surgical interventions after the course of antibiotics for aorta vascular graft infection with favorable outcomes.^8,9)^ However, there are no clear criteria for the timing of radical surgery. We administered a complete course of antibiotics before performing surgery in cases 4 and 5, resulting in their infection being under control at the time of surgery. Conversely, because most cases of AEnF present with bleeding without active signs of infection, early surgery would prevent the onset of infection in these patients. Accordingly, we suggest early surgical intervention with the two-stage strategy for AEnF and radical surgery after a course of antibiotics for AEnE.

Regarding the surgical technique, complete removal of an aortic aneurysm with the preexisting graft, along with nonanatomical revascularization, such as axillary-femoral bypass, was used to be widely performed. However, the outcomes of this procedure were not favorable, with poor long-term patency of the bypass, disruption of the aortic anastomosis, and early mortality.^9)^ Recently, total or partial replacement of the aorta using prosthetic grafts or autologous veins, which results in more physiological and anatomical revascularization, is becoming more common.

In this procedure, total replacement is ideal in view of the potential exposure of the graft to intestinal juices, although it is unattainable in some cases because the entire graft cannot be exposed without injuring adjacent tissues. We performed partial replacement without resecting strong adhesions for all patients in this report, and four of them have not developed infectious complications so far. We think that replacement as much as possible can be the effective method.

Different treatment strategies should be used for AEnF versus AEnE. Specifically, patients with AEnF should undergo emergent endovascular abdominal aortic aneurysm repair to achieve hemodynamic stability, followed by radical open surgery at a later time. By contrast, patients with AEnE should first be treated with a course of antimicrobial therapy and should then undergo radical open surgery.

## Conclusion

We reported five cases of sAEF following OAR for aneurysm repair. In our series, timely management of bleeding and infection by surgical intervention resulted in good outcomes.
